# Development of patient- and observer-reported outcome measures to assess COVID-19 signs and symptoms in children and adolescents

**DOI:** 10.1186/s41687-023-00542-6

**Published:** 2023-01-26

**Authors:** Carla Romano, Margaret Mayorga, Javier Ruiz-Guiñazú, Géralyn C. Trudel, Sheri Fehnel, Kelly McQuarrie, Eric K. H. Chan, Eva G. Katz

**Affiliations:** 1 RTI Health Solutions, Research Triangle Park, NC USA; 2 Janssen Research and Development, Beerse, Belgium; 3 Janssen Global Services LLC, Raritan, NJ USA; 4 Janssen Global Services LLC, Horsham, PA USA

**Keywords:** Patient-reported outcome, Observer-reported outcome, COVID-19, Content validity, Pediatric, Adolescent, Caregiver

## Abstract

**Background:**

The Symptoms of Infection with Coronavirus-19 (SIC) is a 30-item patient-reported outcome measure to evaluate the presence and severity of COVID-19 signs/symptoms in adults. This study expanded the context of use of the adult SIC among adolescents aged 12–17 years and supported a pediatric adaptation (the Pediatric SIC [PedSIC]) for caregiver assessment of signs/symptoms in children aged < 12 years.

**Methods:**

Draft versions of the PedSIC and reference materials containing sign/symptom definitions for adolescents, based on an assessment of the reading level of SIC items by a professional linguist, were developed to facilitate accurate completion of the SIC by adolescents and observer-report (PedSIC) by caregivers. For adolescents, reference materials were intended to provide definitions for selected signs/symptoms identified to have a higher reading level. Iterative rounds of cognitive debriefing interviews were conducted from November 2020 to January 2021 to evaluate adolescent understanding of the SIC reference materials and inform refinement of the PedSIC for caregivers of children too young to reliably self-report. Participants were identified via databases of individuals who previously expressed interest in participating in qualitative research and were then screened for eligibility. Recruitment quotas were established to improve sample diversity. Thematic analysis and descriptive statistics were used to assess qualitative and demographic data, respectively.

**Results:**

Nine healthy adolescents (mean [SD, range] age, 14 [1.76, 12–17] years, 56% female, 22% non-White; round 1, n = 6; round 2, n = 3) and 17 caregivers (mean [SD, range] age, 34 [6.28, 26–41] years, 59% female, 35% non-White; round 1, n = 9; round 2, n = 8) were interviewed. Adolescents understood the majority of signs/symptoms (22 of the 30 SIC items) without assistance or use of the reference materials during the cognitive debriefing interview. Definitions were added to the reference materials for 5 additional items, and clarifications provided to existing definitions for 3 items. Seven observer-report (PedSIC) items were modified following feedback from caregivers of healthy young children. Reference materials (similar to those for adolescent use) were developed to support caregiver understanding of the intent of the PedSIC items collecting input from children ages ≥ 5– < 12 years.

**Conclusions:**

Results support using the SIC, PedSIC, and their associated reference materials to evaluate the presence and severity of COVID-19 signs/symptoms in adolescents and children aged < 12 years via caregiver-supported report, respectively.

**Supplementary Information:**

The online version contains supplementary material available at 10.1186/s41687-023-00542-6.

## Background

As of October 2022, there have been > 617 million confirmed cases of coronavirus disease 2019 (COVID-19) worldwide, with > 6.5 million COVID-19-related deaths to date [1]. In the United States, the Centers for Disease Control and Prevention (CDC) has reported > 95 million cases of COVID-19 and > 1 million deaths [2]. During the same period, > 14.0 million children in the United States contracted COVID-19, with > 1,300 COVID-19–related deaths reported in children aged 0–17 [3, 4]. Vaccination rates among children aged 5–11 years remain low in the United States, with 39% having received 1 dose of a COVID-19 vaccine, 32% having completed a primary vaccine series, and only 15% having received a first booster dose [5]. In the adolescent population aged 12–17 years, 71% have received at least one dose and 61% have completed a COVID-19 vaccination series, but only 29% have received a first booster dose [5]. Data on global rates of vaccination in children are evolving with the approval of vaccines in pediatric populations [6].

Although children may generally experience asymptomatic infection or milder disease compared with adults, they can experience a variety of symptoms [7, 8]. COVID-19 symptoms may resemble other respiratory diseases and include fever or chills, cough, shortness of breath or difficulty breathing, fatigue, muscle or body aches, sore throat, and congestion or runny nose; unique COVID-19 symptoms may include eye problems, headache, nausea or vomiting, diarrhea, skin rash, new loss of taste or smell, and discoloration of fingers or toes [7, 9, 10]. Children and adolescents with underlying medical conditions are at increased risk for severe illness [11, 12]. Children may also have increased incidence of potentially serious conditions following a COVID-19 diagnosis [13], such as multisystem inflammatory syndrome in children (MIS-C), a rare condition requiring intensive care in a significant proportion of patients [11, 14]. Moreover, like adults, children and adolescents can develop long-term consequences of COVID-19 (termed “long” COVID) [15]. Clinical manifestations and outcomes of COVID-19 may change with the emergence of new variants. Risks of severe outcomes such as emergency department visits and intensive care unit admission in children were lower with the Omicron variant compared with the Delta variant [16, 17]; however, upper respiratory infections increased during predominance of the Omicron variant [18], as did overall COVID-19–related pediatric hospitalizations, particularly in unvaccinated children, owing to the increased transmissibility of Omicron leading to increased numbers of infected individuals [19–21]. Although several studies have described clinical characteristics and risk for severe illness in children [22–24], there remains a need for prospective studies of signs and symptoms of COVID-19 in the pediatric population to facilitate proper management and characterization of long-term outcomes [25].

Clinical outcome assessments (COAs), including patient-reported outcome (PRO) measures, provide means to obtain otherwise unattainable assessments of the patient experience and can be used to monitor initial infection, disease progression, and response to treatment [26, 27]. In order to reliably gather these important data, PRO and observer-reported outcome (ObsRO) measures for the pediatric population are necessary [23, 25, 28]. Adolescents aged 12–17 years are typically able to reliably self-report signs, symptoms, and impacts of disease using age-appropriate measures; younger children may not have the ability to read, understand, and fully self-report [28]. Observer assessment is recommended for very young children (< 5 years of age) who are not able to reliably report their own health status [28], but limits the report to observable signs alone. At the time of this study, no COVID-19–specific tools existed for self-completion by adolescents or observer report for young children [27].

The Symptoms of Infection with Coronavirus-19 (SIC) is a PRO measure that uses a checklist approach to assess the presence and severity of signs and symptoms specific to COVID-19 in adults [29] and could be used to monitor for infection. In this prospective, observational, qualitative research study, we: (1) evaluated the understanding of the SIC and accompanying adolescent reference material by adolescents and (2) developed the pediatric SIC (PedSIC) and caregiver reference materials to facilitate observer report. Materials were optimized based on input from healthy adolescents and caregivers of healthy young children, respectively.

## Methods

### Symptoms of infection with Coronavirus-19

The SIC includes 30 items marked as present or absent, 25 of which are followed by a numerical rating of severity. Concept elicitation for the SIC with input from adult COVID-19 patients and caregivers has been previously described [29]. A reading level analysis of the SIC was conducted by a professional linguist using the Lexile Framework for Reading analyzer (2021). The analyzer scores reading level in quartiles, and the grade level assigned was based on the Lexile score for the 50th percentile of children. The SIC items ranged from third-grade to sixth-grade reading levels, and the SIC instructions were assigned a ninth-grade level (Additional file 1: Table S1). Overall, the SIC reading level was considered appropriate for adolescents. After assessment of the SIC reading level, reference materials were developed by professional linguists and COA experts to provide definitions of signs and symptoms of COVID-19 that exceeded the age-appropriate reading level for adolescents aged 12–17 years. The initial draft of the reference card included definitions for 13 SIC items and was limited to a single page for ease of use. In this study, adolescents aged 12–17 years provided feedback on the SIC and accompanying reference card during two iterative rounds of interviews. The SIC reference card was then refined and optimized with the goal of ensuring that the SIC could be easily understood and completed by adolescents aged 12–17 years.

### Pediatric symptoms of infection with Coronavirus-19

The PedSIC is a version of the SIC developed for children. While the PedSIC may be used by adolescents aged 12–17 years if indicated to be more appropriate (ie, limited reading ability or concomitant issues that limit the ability to self-report), this instrument was developed primarily as an ObsRO measure to facilitate caregiver reporting of COVID-19 signs and symptoms in children aged < 12 years. Symptoms were identified through a targeted literature review, including CDC recommendations of signs and symptoms to monitor in young children with review by infectious disease experts. The PedSIC includes one section for completion by caregivers based on observations alone (including 15 signs of COVID-19 assessed using a 5-point verbal rating scale) and a second section for completion by caregivers with input from children aged > 2 years old to allow for the most accurate reporting where observations alone may not be sufficient. In this study, two iterative rounds of interviews were conducted with caregivers of healthy young children to optimize the PedSIC instructions and items. After the first round of interviews, internal clinical experts offered suggestions during a web-assist video conference held to improve clinically meaningful elements of the PedSIC and SIC adolescent reference card and inform revisions of the items based on emerging data around the patient experience of COVID-19. After the completion of the first round of caregiver interviews, a reference card was developed to support caregivers in fully understanding the intent of each item in the second section of the PedSIC. The reference card was drafted using feedback gathered during caregiver interviews coupled with information from internal experts regarding specific intent of each item, providing caregivers with sample descriptions of words or language so that they could explain each question in language familiar to the child.

### Participants

Data were collected in individual interviews with healthy adolescents aged 12–17 years for the SIC and caregivers of healthy children aged < 12 years for the PedSIC; all participants were in the United States. Additional eligibility criteria included a willingness and ability to participate in a 1-h interview via telephone or online system; the ability to read, speak, and understand English; parental permission and a willingness and ability of adolescents to provide assent; and the ability of both adolescents and caregivers to access documents electronically. Participants did not report history of COVID-19 or current diagnosis of SARS-CoV-2 infection. Participants were recruited by third-party qualitative research firms (Reckner Healthcare, Chalfont, PA or L&E Research, Raleigh, NC) via databases of individuals who had previously expressed interest in participating in qualitative research. A convenience sampling approach was used for both adolescents and caregivers. All adolescents assented and all caregivers provided informed consent. RTI International’s institutional review board (Research Triangle Park, NC) reviewed the study materials and provided approval for this research in adolescents. Caregiver interviews were exempt from formal review. Recruitment targets were set to improve sample diversity of adolescents and caregivers regarding sex, age, and race/ethnicity, and for caregivers only, education level.

### Interviews

Interviews were conducted from November 2020 through January 2021. To reduce the potential for interviewer bias, the research team alternated roles between interviewer and note-taker and utilized a semi-structured interview guide. Additionally, the initial development of the adolescent reference card was led by an independent linguist who was not part of the interview team. Interviews were audio recorded and transcribed and the transcripts were de-identified prior to analysis. Each interview began with a study overview followed by a detailed cognitive debriefing of the SIC/PedSIC. Cognitive debriefing focused on understanding participant thought processes as they responded to each item in the PRO/ObsRO measure, and targeted probes were utilized following a semi-structured interview guide to elicit these details. Adolescents provided feedback on the definitions in the draft reference card. In addition to the PedSIC, caregivers were also asked to provide feedback for two items addressing COVID-19 symptom status and change, as well as one item regarding observation of medically emergent symptoms. To facilitate refinement of the measures and reference materials, the interviews were conducted in two iterative sets. Interviewers also posed follow-up questions designed to further elucidate the participants’ question-answering process, and to identify any revisions to the reference materials that could enhance understanding of the SIC/PedSIC instructions, questions, and/or response options.

#### Adolescents

**Fig. 1:**
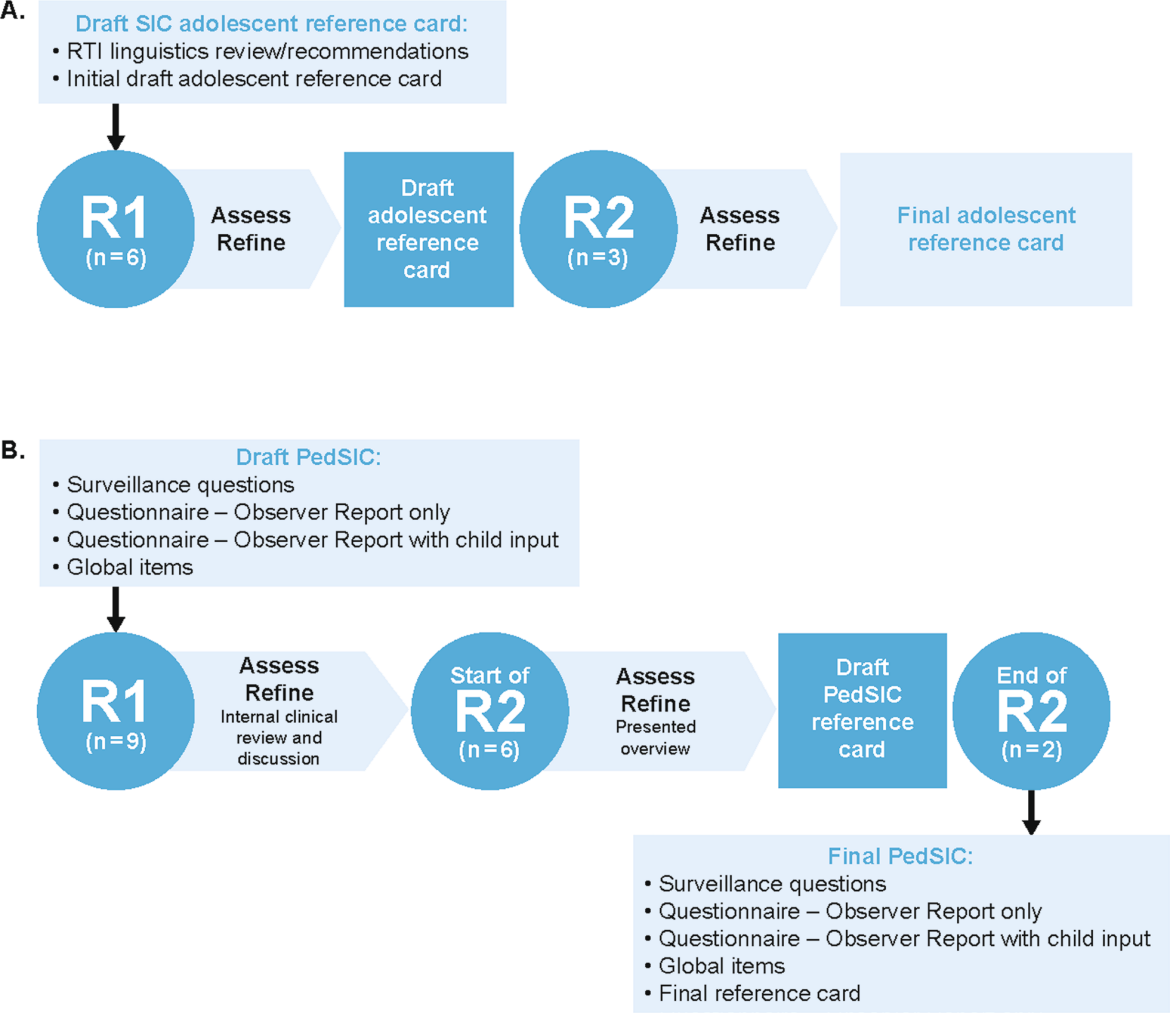
Cognitive debriefing process for **A** adolescents and **B** caregivers. In panel B, surveillance questions refer to an item designed to monitor children for initial infection and medically emergent signs and symptoms of COVID-19. PedSIC, Pediatric Symptoms of Infection with Coronavirus-19; SIC, Symptoms of Infection with Coronavirus-19

Figure 1A shows the iterative interview process (rounds 1 and 2) with adolescents to assess the relevance, clarity, and comprehensiveness of the SIC and associated reference materials and refine the draft content of the materials. Adolescent participants were asked about the SIC instructions, definition of the “recall period,” SIC items, and the use of the reference card to facilitate understanding of each item of the SIC. Upon completion of the first set of interviews (n = 6), findings were reviewed via field notes, and changes to the SIC reference card were made before conducting the second round of interviews (n = 3).

#### Caregivers

Rounds 1 and 2 of the interview process with caregivers are shown in Fig. 1B. During cognitive debriefing, participants reviewed each of the measures together with the interviewer either using a paper copy of the items that was e-mailed to each participant in advance and/or using a web link for joint viewing. Participants were asked for their input on the COVID-19 surveillance (monitoring for infection or emergent symptoms) and instructions, recall period, symptoms described and their severity during the 24-h recall period, fever/temperature, observer-report with child input, global impressions of severity, and patient global impression of change. Upon completion of round 1 of interviews (n = 9), appropriate changes were made to the PedSIC before conducting the second round of interviews (n = 8). A PedSIC completion reference card to aid caregivers in collecting child input was developed near the end of round 2 of the interviews.

### Data analysis

Standard qualitative methods were applied using an inductive approach to analyze the data collected during the individual interviews. Specifically, a thematic approach aided by field notes and interview transcripts was used to analyze the results of the interviews [30]. All analyses were conducted by the two interviewers. To assess potential issues with the PedSIC measure, interviewers explored participant understanding of instructions, items, and response scales to determine if barriers, such as technical terminology or confusing instructions, impaired participant comprehension, completion of the measure, or application of the reference materials. A similar approach was used to assess adolescent completion of the SIC and ability to use the reference card. Descriptive statistics were generated for demographic variables and reported in aggregate; no inferential statistical analyses were conducted. Neither a formal coding guideline nor a formal assessment of concept saturation was required, given the focus on cognitive debriefing of the measure. Any issues identified during interviews were addressed directly in either the PedSIC measure or reference cards and tested in subsequent rounds. The results of analyses were used to inform revisions throughout the iterative testing process.

## Results

### Demographic data

Demographics for healthy adolescent participants (n = 9) were recorded at screening (Table 1). Round 1 consisted of interviews with mostly female (n = 5), White adolescents, and round 2 consisted of male adolescents (n = 3) of White, African American/Black, or mixed heritage. The mean age of adolescent participants was 14 years (SD, range: 1.76, 12–17 years); 56% of participants were in middle school (12–13 years), with the remainder in high school (14–17 years). Demographic characteristics reported at screening for the 17 caregivers and a summary of the age of the children that were the focus of the interviews are presented in Table 2. Overall, 59% of respondents were female, 36% were non-White, and education ranged from high school through a postgraduate degree. The mean age of caregivers was 34 years (SD, range, 6.28, 26–41). Five caregivers (29%) had more than one child; therefore, they were asked to focus on one child (based on age recruitment targets) for completion of the ObsRO measure. The children ranged in age from newborn to 11 years.

**Table 1:** Demographic Characteristics Reported at Screening of Healthy Adolescents Aged 12–17 Years

Characteristics, n (%)	Round 1 (n = 6)	Round 2 (n = 3)	Total (N = 9)
*Sex*			
Male	1 (17)	3 (100)	4 (44)
Female	5 (83)	0 (0)	5 (56)
*Current age, mean years (range)*	14.17 (12–17)	14.00 (13–16)	14.11 (12–17)
Aged 12– < 15 years	4 (67)	2 (67)	6 (67)
*Race*			
White	6 (100)	1 (33)	7 (78)
African American/Black	0	1 (33)	1 (11)
Mixed	0	1 (33)	1 (11)
*Education*			
Middle school (grade 6–8)	3 (50)	2 (67)	5 (56)
High school (grade 9–12)	3 (50)	1 (33)	4 (44)

**Table 2:** Demographic Characteristics Reported at Screening of Caregivers of Children Aged < 12 Years

Characteristics, n (%)	Round 1 (n = 9)	Round 2 (n = 8)	Total (N = 17)
*Sex*			
Male	4 (44)	3 (38)	7 (41)
Female	5 (56)	5 (63)	10 (59)
*Current caregiver age, mean years*	35.00	32.50	34.00
Race*			
White	5 (56)	6 (75)	11 (65)
African American/Black	3 (33)	1 (13)	4 (24)
Asian	0	1 (13)	1 (6)
Other	1 (11)	0	1 (6)
Hispanic (yes)	2 (22)	0	2 (12)
*Education, n (%)*			
High school/GED	0	1 (13)	1 (6)
Some college	4 (44)	2 (25)	6 (35)
College degree	3 (33)	3 (38)	6 (35)
Postgraduate	2 (22)	2 (25)	4 (24)
*Children’s age, n*			
Newborn–5 months	0	3 (38)	3 (18)
6–12 months	2 (22)	0	2 (12)
13–35 months	3 (33)	2 (25)	5 (29)
36 months–6 years	2 (22)	2 (25)	4 (24)
7–11 years	2 (22)	1 (13)	3 (18)

*Total for race exceeds 100% due to rounding

### Healthy adolescents (SIC and reference materials)

Overall, adolescents interpreted the instructional text for the SIC and reference card as intended. In both rounds of interviews, the “recall period” was consistently interpreted correctly, ie, the time that occurred in the 24 h immediately preceding beginning self-completion of the SIC. For example, if the participant was reading the instructions in the morning, this would include recalling symptoms back to the same time the prior morning. Participants also generally reported that recalling the prior 24 h would be easy when completing the SIC. Participants in both rounds of interviews found the checklist approach of the SIC easy to use, with clear response options. The use of the 11-point numerical rating scale for severity of 0 (none) to 10 (worst possible) on the SIC was also assessed in both rounds of interviews; adolescents found the scale clear, easy to use, and familiar.

Overall, participants felt that the SIC was generally clear and that it would be easy to complete electronically on a daily basis; a few younger participants preferred paper over electronic versions, noting paper would be more readily accessible. Participants easily understood 22 of the 30 items, and feedback supported retention of the reference definitions for those items without modification. With respect to the remaining 8 items, definitions for 4 concepts (“cough,” “chest congestion,” “fever,” and “red or bruised looking feet or toes”) were added to the reference card after round 1. For example, although adolescents were familiar with the concept of “cough,” they were unsure how to qualify it in terms of frequency, severity, or type. After receiving feedback in round 1, a definition was added to the reference card stating “cough” could include dry or wet coughing. Clarifications to 3 existing definitions on the reference card (“problems thinking clearly/brain fog,” “feeling faint,” and “skin rash”) were made after round 1 to improve understanding. A full description of “wheezing” was added to the reference card in round 1, with further modification in round 2 to clarify the item. Representative quotations from adolescents regarding individual understanding of the SIC are presented in Additional file 1: Table S2.

### Caregivers of healthy young children (PedSIC and reference materials)

The first question of the PedSIC was designed to monitor children for initial infection as well as medically emergent signs and symptoms of COVID-19 (“new or worsening of symptoms or health concerns”). New symptoms were described as changes in health status that caregivers had not previously noticed, whereas worsening symptoms were defined as something that had been affecting the child but had increased in severity over time. This first question and accompanying instructions regarding monitoring for infection and medically emergent COVID-19 signs and symptoms were generally well-received by participants, who indicated that they understood the intent of the question (ie, to capture health concerns that may require care). Clarifications were made to the text of this item, including removal of the phrase “health concerns” which was found to be potentially redundant. After round 1, other items were updated for clarity based on participant and expert feedback. For example, “trouble breathing (increased work of breathing),” was simplified to “trouble breathing.” Additionally, clinical experts suggested including “bloodshot or very red eyes,” and multiple items to capture gastrointestinal symptoms. In round 2, participants understood the additional gastrointestinal symptoms; however, symptoms describing confusion and somnolence were further revised.

Interviewees consistently reported the PedSIC instructional text, recall period, and response options for the observer-report sections to be clear. The items addressing “decreased activity,” “irritability (examples: crying, not easily soothed),” “shortness of breath (difficulty breathing),” “wheezing (whistling sound),” “chest congestion (mucus in chest),” “chills,” and “eye irritation/discharge (examples: eyes appear red or swollen, watery or itchy eyes, discharge coming out of eyes)” were modified following feedback received during both rounds of interviews. Representative quotations from caregivers regarding individual understanding of the PedSIC are presented in Additional file 1: Table S3.

All participants found the instructions for the observer-report with child input component of the PedSIC to be clear and easy to understand. After reviewing specific items, all participants indicated they would likely need to rephrase the questions or otherwise provide additional information to their children to elicit an accurate response for each question. Sample descriptions for 11 items were included on a reference card created for caregivers with standardized instructions to read to their children to capture child input the most accurately. For example, the sample description “[point to or touch outside of ear] Do your ears hurt on the inside?” was included for the item “Do your ears hurt?” Clarifications were made to the following items using the child input reference card: “Do you feel sick to your stomach/belly?”; “Can you smell things like usual?”; “Can you taste things like usual?” Caregivers of children aged ≤ 4 years discussed potential difficulty for their child to accurately report on symptoms they had not yet experienced. Thus, based on caregiver feedback, it was determined that the PedSIC with child input could be administered most reliably to children aged 5 years and older. Notably, following the inclusion of the reference language, caregivers expressed greater confidence that younger children could accurately respond.

## Discussion

Cognitive debriefing is an important tool to evaluate patient understanding of the descriptive words or phrases generated during concept elicitation and respondent question/answer thinking process that serve as the foundation for any given COA [31]. By conducting cognitive debriefing interviews, the content validity of a PRO measure can be supported by assessing the comprehension of the measure in the target population, ensuring it is interpreted as intended by the developer, covers appropriate concepts, and does not require specialty training [31]. In this study, we provided evidence to support the SIC and PedSIC by describing feedback obtained from adolescents and caregivers via cognitive debriefing. Through this process, we confirmed the need for reference materials to facilitate self-completion of the SIC by adolescents. The completion reference card was assessed through qualitative interviews and further refined to meet the needs of adolescents with reading levels potentially lower than the US sixth grade. All adolescents in the study found the reference card to be a useful resource and with few exceptions, were able to read and respond to each SIC item without it.

The PedSIC uses a combination of observer and observer–child reports to capture “best available” data for children younger than age 12, including signs of infection that are clearly observable and reportable by a child’s caregiver and symptoms that incorporate perspectives directly from the child. Caregiver interviews supported both the content and validity of the PedSIC, with the use of a reference card to facilitate communication with their child and subsequent completion. Reference materials were optimized in this study to support standardized administration of the PedSIC. The minimum age for child input was raised to 5 years as a result of the caregiver interviews.

Currently, there are gaps in research with respect to differential phenotypes of COVID-19 in the pediatric population compared to adults, outcomes in children with underlying medical conditions, and children with long COVID-19 [25]. COAs may help monitor disease progression and facilitate long-term follow up of patient health-related quality of life (HRQoL), as well as inform clinical trial design [27]. The adult SIC was developed to align with current Food and Drug Administration (FDA) guidance, patient-focused drug development guidance, and published best practice recommendations regarding use of PRO measures in clinical trials and characterization of the patient experience of COVID-19 [29, 32–36]. The FDA guidance stipulates that any COA used to collect patient experiences or to be referenced in product labeling must be developed with extensive input from patients in the population of interest and thoroughly psychometrically evaluated in the target population. Consistent with these guidelines, the measures evaluated in this study were developed and supported by gathering input of caregivers and adolescents to optimize the adaptations.

Limitations of this study include a lack of greater ethnic and racial diversity of participants and a relatively small sample size (n = 9 adolescents; n = 17 caregivers); only 3 adolescents participated in round 2, which is fewer than the number of participants recommended for qualitative research [37]. Participants were also limited to the United States, meaning the measures evaluated in this study would require translation and cultural adaptation for use in global populations [38]. Furthermore, interviewed participants (and/or their child) did not report history of or current diagnosis of SARS-CoV-2 infection; therefore, we were unable to assess self-completion when participants were sick or experiencing symptoms. Psychometric validation in pediatric and adolescent patients with COVID-19, including those with ongoing symptomatic COVID-19 and post–COVID-19 syndrome [39], is necessary to establish reliability, responsiveness, known-groups validity, and meaningful change thresholds of the measure.

This study supports the extension of the context of use of the SIC in pediatric and adolescent populations. To our knowledge, no PRO or ObsRO measures have been developed to capture the symptomatic experience of COVID-19 in children and adolescents; therefore, the SIC, PedSIC, and reference materials for each fulfill an important unmet need.

## Conclusions

Broader application of the SIC and implementation of the PedSIC will provide appropriate measures for the collection of signs and symptoms of COVID-19 in adolescent and younger pediatric populations for both vaccine and treatment trials. Quantitative assessment to demonstrate the psychometric properties of the PedSIC in patients with COVID-19 is needed.

## Supplementary Information

**Additional file 1: Table S1**: Lexile Framework Reading Level Assessment of SIC Items. **Table S2**: Representative Descriptions of Adolescent Feedback of SIC Items. **Table S3**. Representative Descriptions of Caregiver Feedback of PedSIC Items.

## Data Availability

Data are available upon reasonable request.

## Abbreviations

CDC: Centers for Disease Control and Prevention
COA: Clinical outcome assessment
COVID-19: Coronavirus disease-2019
FDA: Food and Drug Administration
HRQoL: Health-related quality of life
MIS-C: Multisystem inflammatory syndrome in children
ObsRO: Observer-reported outcome
PedSIC: Pediatric Symptoms of Infection with Coronavirus-19
PRO: Patient-reported outcome
SARS-CoV-2: Severe acute respiratory syndrome coronavirus 2
SD: Standard deviation
SIC: Symptoms of Infection with Coronavirus-19
