# Supplementary material for: Development of patient- and observer-reported outcome measures to assess COVID-19 signs and symptoms in children and adolescents
Source: J Patient Rep Outcomes. 2023 Jan 26;7:7. doi: 10.1186/s41687-023-00542-6 (PMC9879250; doi:10.1186/s41687-023-00542-6)
Supplement: Supplementary file 1 — Additional file 1: Table S1: Lexile Framework Reading Level Assessment of SIC Items. Table S2: Representative Descriptions of Adolescent Feedback of SIC Items. Table S3. Representative Descriptions of Caregiver Feedback of PedSIC Items. [file 41687_2023_542_MOESM1_ESM.docx]

**Supplementary Material**

**Supplementary Table 1.** Lexile Framework Reading Level Assessment of SIC Items

| **US Grade level** | **Item** |
| --- | --- |
| 3^rd^ grade | - Cough - Runny nose - Sneezing - Skin rash - Diarrhea - Vomiting - Fever - Uncontrollable body shaking/shivering - Decreased sense of smell - Decreased sense of taste - Red or bruised-looking feet or toes |
| 4^th^ grade | - Feeling generally unwell (run down) - Fatigue (tiredness) - Physical weakness - Sore throat - Chest pain/pressure/tightness - Muscle aches/pains - Joint aches/pains - Headache - Feeling faint - Problems thinking clearly/brain fog - Chills - Eye irritation/discharge - Nausea - Abdominal/stomach pain - Loss of appetite |
| 6^th^ grade | - Shortness of breath (difficulty breathing) - Nasal congestion - Wheezing (whistling sound while breathing) - Chest congestion (mucus in chest) |
| 9^th^ grade | - Instructional text |

**Supplementary Table 2.** Representative Descriptions of Adolescent Feedback of SIC Items

| **Item** | **Description** | **Interview Round** |
| --- | --- | --- |
| Cough | “[Would you include dry cough] *I'm not sure because it doesn't really say specifically what type of cough you guys are looking for. I feel like you could define that a little more on the card. Just like say what type of cough and yeah. Without any specific directions I would think personally that it would just be how severe was your basic, like any type of cough that there is, meaning dry, really anything…I think you can maybe add something to like the reference card about that because maybe someone doesn't really know what the definition of a wet cough or a dry cough is.”* | 1 |
|  | *“When you say coughing, do you mean coughing a lot or like the cough hurt?”* | 1 |
| Wheezing | *“I don't think I've ever had that. And I was a little confused, to be honest. I'm not sure what a whistling sound while breathing sounds like. But I think I can put a pretty clear picture in my head with the explanation I was just a little confused.”* | 1 |
|  | *“I'm not going to lie, I honestly don't even know because I really don't know what causes wheezing or what makes you whistle when you breathe…So if you were to put the reason why it happens, that would be helpful. [blockage in your airway or a problem with your airway, how about problem with your airway?] Yeah, that may be more understandable.”* | 2 |
| Problems thinking clearly/brain fog | *“I'm not really sure what you mean by that, but the problem thinking clearly, I guess that’s not able to think properly in the moment. I think that [the reference card] definitely helps…Maybe you could use, like, a different word than clearly…it might be good to have another word there just other than clear, because you’re saying that a lot in this.”* | 1 |
| Skin rash | *“If you're just asking about all over your body or in general, like, oh, if you have a skin rash on your arm, that might be a symptom of COVID than if you have a skin rash on your leg or something?”* | 1 |

**Supplementary Table 3.** Representative Descriptions of Caregiver Feedback of PedSIC Items.

| **Item** | **Description** | **Interview Round** |
| --- | --- | --- |
| Decreased activity | *“…actually, because now he’s at the stage where he should be awake a few more times a day or a couple more hours, you know within the hour or 2. That, I could notice decreased activity, if he’s sleeping more throughout the day.”* | 2 |
| Irritability | *“So, for him when he is irritable, he’s not always just crying…But he will sometimes, like, throw thing or just, you can’t tell what he wants. Does he want food? Does he want a drink? Does he want a toy? You try to give him all the things he wants and he is throwing them and he is still, you know, upset. Not necessarily crying…So, I would say that’s also irritable.”* | 1 |
|  | *“In my baby, crying, like it said, not easily soothed or consoled. Not wanting to nurse.”* | 2 |
| Shortness of breath | *“In my son, not necessarily the shortness of breath but the difficulty breathing. Like I said, his respiratory rate. How hard he was trying to take deep breaths. Things like that. Personally, shortness of breath is more of a description as older people would say, ‘Oh man, I’m a little short of breath.’ That would be a way that they described their breathing.”* | 1 |
| Chest congestion | *“I don’t know that I could observe chest congestion in an infant. Yeah. He’s never coughed up anything, or you know, I don’t know if that applies to that age group.* [Coughing up mucus would be more observable] *Yeah. I mean, I guess that’s more physical, right, whereas I read chest congestion, mucus in the chest, I think of hearing a sound.”* | 1 |
|  | *“They’re coughing up mucus, yes, I would agree with this one as well, only though if they’re able to cough it up. I’m not sure that a lot of people would be able to tell if there’s actually mucus in the chest, unless it’s separate and linked back to the wheezing.”* | 2 |
| Chills | *“Chills, I will probably put in parenthesis, ‘Shaking.’…I use that word a lot when he shakes. Shivering. That’s a good one.* | 1 |
